# Quality of life among lower limb prosthetic users in Jordan: a cross-sectional study using the Arabic SF-36

**DOI:** 10.3389/fresc.2025.1665006

**Published:** 2025-09-03

**Authors:** Mahmoud Alfatafta, Huda Alfatafta, Amneh Alshawabka, Huthaifa Atallah, Anthony McGarry

**Affiliations:** ^1^ Department of Prosthetics and Orthotics, School of Rehabilitation Sciences, The University of Jordan, Amman, Jordan; ^2^ Doctoral School of Health Sciences, Faculty of Health Sciences, University of Pécs, Pécs, Hungary; ^3^ Biomedical Engineering, The University of Strathclyde, Glasgow, United Kingdom

**Keywords:** lower limb amputation, quality of life, SF-36, prosthetic users Jordan, healthrelated outcomes, rehabilitation, cross-sectional study

## Abstract

**Introduction:**

Lower limb amputation is a life-altering event that affects multiple dimensions of quality of life (QoL), including physical functioning, emotional well-being, and social participation. Despite the clinical importance of QoL assessment in prosthetic rehabilitation, few studies have examined the multidimensional impact of amputation and prosthesis use in the Jordanian context. This study aimed to evaluate the QoL of lower-limb prosthetic users in Jordan and examine potential differences based on gender, and amputation level.

**Methods:**

A cross-sectional study was conducted with 293 adults with lower limb amputations, using prostheses. Participants completed the Arabic version of the RAND 36-Item Health Survey (SF-36). Data were collected from public and private rehabilitation centers across Jordan. Eight QoL subscales were scored on a 0–100 scale. Descriptive statistics, group comparisons (gender and age group), and multiple linear regression were conducted to assess predictors of QoL.

**Results:**

The highest domain scores were observed in Emotional Well-being (median = 77.0, IQR 55.0–90.0) and Social Functioning (median = 100.0, IQR 62.5–100.0), while the lowest were in Role Physical (median = 50.0, IQR 0.0–100.0) and General Health (median = 41.7, IQR 33.3–58.3). Group comparisons revealed significant differences by amputation level in Role Physical, Role Emotional, and Composite QoL scores, with individuals with more proximal amputations reporting lower outcomes. Regression analyses showed that older age significantly predicted poorer Physical Functioning (*β* = –0.75, *p* < 0.001), and male participants scored higher than females in the same domain (*β* =  + 8.67, *p* = 0.0227). Amputation level was significantly associated with QoL in select domains in group comparisons, though it was not a significant predictor in multivariable regression. Education level was not a significant factor in either analysis. The models explained a modest proportion of variance, with *R*² values ranging from 0.03 to 0.19 across SF-36 domains.

**Conclusions:**

Lower limb prosthetic users in Jordan experience moderate impairments in physical QoL domains, particularly among older adults. Emotional and social domains were relatively preserved. Demographic factors, especially age and gender were associated with differences in specific QoL outcomes and should be considered in the development of personalized rehabilitation strategies.

## Introduction

Lower limb amputation is a life-altering event with long-term physical, psychological, and social consequences. Globally, the leading causes of lower limb amputation include diabetes mellitus, peripheral vascular disease, trauma, malignancies, and infections (1). As diabetes and road traffic injuries increase in low- and middle-income countries, the burden of limb loss is expected to rise significantly in regions such as the Middle East and North Africa (MENA) (2). Jordan, like many countries in the region, faces an increasing prevalence of diabetes-related complications and traumatic injuries, yet comprehensive data on outcomes after amputation remain scarce (3).

Beyond the immediate surgical and physical recovery, amputation affects multiple domains of a person's life. These include mobility, independence, social integration, emotional well-being, body image, employment, and access to healthcare services (3, 4). To address the complex, multidimensional impact of limb loss, the measurement of health-related quality of life (HRQoL) has become central in evaluating rehabilitation outcomes. It is increasingly recognized that functional independence alone does not capture the lived experience of prosthetic users; emotional adaptation, social participation, and subjective well-being are equally important indicators of successful rehabilitation (5, 6).

One of the most widely used instruments to assess HRQoL is the Form-36 Health Survey (SF-36), a validated, multidimensional tool that evaluates eight key domains: physical functioning, role limitations due to physical or emotional problems, bodily pain, general health, vitality, social functioning and emotional well-being (7, 8). It provides a comprehensive profile of health status and has been adapted and validated in many languages, including Arabic (9, 10). The Arabic version of the SF-36 has demonstrated good reliability and internal consistency in several Arab-speaking populations and is suitable for use in both clinical and research settings (10–12).

Multiple studies have explored the factors associated with HRQoL in lower limb amputees (13). Physical function is often reported to be significantly reduced, especially among individuals with higher-level amputations (e.g., transfemoral) or limited access to rehabilitation services (9, 14). Emotional well-being and social functioning, however, may vary depending on cultural, familial, and psychosocial support structures (6, 15). Research suggests that men often report better physical and emotional outcomes than women, potentially due to differences in role expectations, physical strength, or access to prosthetic care (16, 17). Educational attainment, age, and prosthetic use are also variably associated with HRQoL outcomes, though the findings are not always consistent across countries and populations (15, 17).

In South Asia and Africa, studies have shown that poor pain management, phantom limb sensations, comorbidities, and unemployment negatively affect both physical and mental health among amputees (15, 18, 19). In contrast, studies in Western settings have found that emotional resilience, optimism, and adaptive goal setting can improve QoL despite high levels of physical impairment (20). However, such psychosocial constructs are rarely assessed in clinical practice, especially in resource-limited or culturally conservative settings.

In Jordan and other Arab countries, there is a lack of large-scale, systematic research on the quality of life of prosthetic users. Rehabilitation services vary widely between urban and rural areas, and users often face delays in prosthesis provision, limited follow-up, and inadequate psychological support (21, 22). Cultural norms and family expectations may further influence emotional adjustment and perceived well-being, but these factors remain under-explored.

Given this gap, the present study aimed to assess the quality of life among lower limb prosthetic users in Jordan using the Arabic version of the SF-36. The study also explored the relationship between demographic and clinical factors including age, gender, educational level, and amputation level and QoL outcomes. It was hypothesized that age, gender, and amputation level would significantly affect SF-36 scores. By identifying the domains most affected and evaluating potential predictors, this study provides new insights that can inform clinical practice, rehabilitation policies, and culturally sensitive interventions in Jordan and similar contexts.

## Methods

### Study design

A cross-sectional analytical study was conducted to assess the health-related quality of life (HRQoL) among lower limb prosthetic users in Jordan. Data were collected between December 2023 to January 2025 from patients attending 14 P&O centers covering public, private, military-affiliated, and NGO-operated facilities. The study followed the Strengthening the Reporting of Observational Studies in Epidemiology (STROBE) guidelines.

A total of 293 adult participants (≥18 years) with unilateral lower limb amputation and current use of a prosthesis for at least 6 months were included. All participants were required to be able to communicate verbally and provide informed consent. Exclusion criteria included bilateral amputations, cognitive impairments, or concurrent severe psychiatric illness. Participants were recruited using convenience sampling during routine clinical visits. While convenience sampling may introduce selection bias, efforts were made to ensure diversity in service providers and patient demographics, enhancing the study's generalizability. This sampling method was chosen due to logistical constraints, variable patient availability, and the need for immediate feedback.

### Data collection

Health-related quality of life was assessed using the Arabic version of the 36-Item Short Form Health Survey (RAND-36), which is based on the SF-36 Version 1. The questionnaire includes eight domains: physical functioning, role limitations due to physical problems, role limitations due to emotional problems, energy/vitality, emotional well-being, social functioning, pain, and general health perceptions. The eight domains were analyzed individually, and Physical Component Summary (PCS) and Mental Component Summary (MCS) scores were not calculated, as the study focused on domain-level findings rather than aggregated summary scores. Responses were transformed into a 0–100 scale according to the official scoring manual, with higher scores indicating better health status (11). The Arabic SF-36 has been validated in multiple Arab-speaking populations with demonstrated reliability (Cronbach's alpha > 0.70 for most domains) (10–12).The survey is available in (RAND Corporation website).

In addition to the SF-36, a demographic and clinical data sheet was used to collect information on age, gender, education level, and amputation level. The average time to complete the questionnaire was approximately 20 min.

Posters were pinned onto notice boards at each provider as well as study adverts that were available as a handout in the waiting areas. Surveys were administered and collected on the same day to ensure consistency in data collection.

Ethical approval for this study was obtained from the Jordanian Ministry of Health, National Ethics Committee for Health Research (MOH/REC/2023/415) and all procedures were conducted in accordance with the Declaration of Helsinki. Informed written consent was obtained from all participants.

### Data analysis

Data were entered and analyzed using IBM SPSS Statistics for Windows, Version 26.0. Descriptive statistics, including means, standard deviations, and frequencies, were used to summarize demographic characteristics and SF-36 subscale scores. Composite QoL score was calculated as the arithmetic mean of the eight SF-36 domain scores for each participant, following approaches used in similar QoL studies (5, 10–12, 15). This single summary measure was used to describe overall quality of life rather than separate physical and mental health components, as our study focused on an integrated QoL perspective.

Data quality was maintained through double data entry and cross-checking to minimize entry errors. All data collectors (research assistants) received standardized training on questionnaire administration to ensure consistency across sites. Missing data were checked at the time of collection, and any incomplete responses were clarified immediately with participants when possible. For the analysis, only fully completed questionnaires were included; no imputation was performed.

The Shapiro–Wilk test was used to assess the normality of SF-36 domain scores, revealing that the data were not normally distributed. Accordingly, non-parametric tests were employed for group comparisons: the Mann–Whitney *U*-test was used to compare scores between males and females, and the Kruskal–Wallis *H* test was used for comparisons across age groups. Chi-square tests were used to examine associations between demographic variables and dichotomized QoL groups (low vs. high composite QoL). Descriptive statistics are presented as medians with interquartile ranges (IQR) rather than means and standard deviations.

Multiple linear regression analysis was performed to examine the effects of age, gender, education level, and amputation level on each SF-36 domain and the composite QoL score. To assess multicollinearity among the independent variables in the regression models, Variance Inflation Factor (VIF) values were examined. All VIF values were below 2.0, indicating no significant multicollinearity and acceptable independence among variables. To reduce the risk of Type I error due to multiple comparisons across SF-36 domains, the Benjamini–Hochberg False Discovery Rate (FDR) correction was applied to all *p*-values derived from group comparisons. This approach controls the expected proportion of false positives while maintaining greater statistical power (23).

Effect sizes were calculated for significant group comparisons using the Mann–Whitney *U* (r) and Kruskal–Wallis (*η*^2^) formulas. Spearman's rank correlation was used to assess intercorrelations among the SF-36 domains. Statistical significance was set at *p* < 0.05 for all analyses.

## Results

A total of 293 participants were included in the study, with a mean age of 48.6 years (SD = 15.9). The majority were male (73.0%), and most had a transtibial amputation (72%). Educational backgrounds varied, with bachelor's and high school degrees being the most common. Table 1 presents the full socio-demographic breakdown.

**Table 1:** Socio-demographic characteristics (*n* = 293).

Variable	Frequency	Percentage
Aged		
<40	96	32.8%
≥40	197	67.2%
Gender		
Male	215	73%
Female	78	27%
Level of amputation		
Transtibial	212	72%
Transfemoral	77	26%
Knee Disarticulation	1	0.75%
Hip Disarticulation	3	1.25%
Level of Education		
Bachelor's degree	84	28.7%
High school	79	27.0%
Secondary school	67	22.9%
Primary school	52	17.7%
PhD	6	2.0%
Master's degree	5	1.7%

The distribution of SF-36 domain scores was assessed using the Shapiro–Wilk test. All domains, including the Composite QoL score, showed significant deviation from normality (*p* < 0.001 for all), indicating non-normal distributions. Accordingly, non-parametric statistical tests were used in all group comparisons.

### Descriptive SF-36 scores

Descriptive statistics were calculated for all eight domains of the SF-36 questionnaire, along with the composite quality of life (QoL) score. Given the non-normal distribution of the data, results are reported using medians and interquartile ranges (IQRs).

The highest median score was observed in Social Functioning (Median = 100.0, IQR = 62.5–100.0), indicating that many participants reported strong social engagement despite limb loss. Emotional Well-being also showed relatively high scores (Median = 77.0, IQR = 55.0–90.0), suggesting preserved emotional health among most respondents. Moderate scores were noted for Vitality (Median = 62.5, IQR = 43.8–87.5) and Pain (Median = 70.0, IQR = 35.0–90.0), reflecting variability in energy levels and discomfort.

Domains related to physical capacity showed lower central values. Physical Functioning had a median of 55.0 (IQR = 30.0–70.0), while Role Physical and Role Emotional both had medians of 50.0 and 66.7, respectively, with wide IQRs spanning the full scale (0.0–100.0), indicating significant variation in perceived limitations. General Health scored the lowest overall (Median = 41.7, IQR = 33.3–58.3), highlighting persistent concerns about health status.

The Composite QoL Score, calculated as the average of the eight domains, had a median of 63.2 (IQR = 44.8–76.2), suggesting moderate overall quality of life among prosthetic users. Participants utilized the full range of response options (0–100), reinforcing the diversity of experiences in this population.

Table 2 provides a complete summary of the descriptive statistics for each domain and the composite score, including median and Interquartile ranges.

**Table 2:** Descriptive statistics of SF-36 domain scores (*n* = 293).

Domain	Median	Interquartile ranges
Physical Functioning	55.0	30.0–70.0
Role Physical	50.0	0.0–100.0
Role Emotional	66.7	0.0–100.0
Vitality	62.5	43.8–87.5
Emotional Well-being	77.0	55.0–90.0
Social Functioning	100.0	62.5–100.0
Pain	70.0	35.0–90.0
General Health	41.7	33.3–58.3
Composite QoL Score	63.2	44.8–76.2

### By gender

The Mann–Whitney *U*-test was used to assess differences in SF-36 domain scores between males and females. Statistically significant gender differences were observed in the Vitality domain (FDR-adjusted *p* = 0.0157) and the Role Emotional domain (FDR-adjusted *p* = 0.0210), with females reporting higher scores in both domains. No statistically significant differences were observed between males and females in the remaining six domains or in the composite QoL score.

### By age group

Participants were categorized into three age groups: <40 years (*n* = 96), 40–60 years (*n* = 127), and >60 years (*n* = 70). The Kruskal–Wallis test identified significant age-related differences in five of the SF-36 domains. Physical Functioning showed the most pronounced difference (FDR-adjusted *p* < 0.0001), followed by Role Physical (FDR-adjusted *p* = 0.0055), Role Emotional (FDR-adjusted *p* = 0.0002), Pain (FDR-adjusted *p* = 0.0002), and Composite QoL score (FDR-adjusted *p* = 0.0002). No significant differences were detected in the domains of Emotional Well-being, Vitality, or Social Functioning across age groups.

### By level of amputation

Participants were categorized into four groups: transtibial (TT), transfemoral (TF), knee disarticulation (KD), and hip disarticulation (HD). The Kruskal–Wallis test was used to compare domain scores of the SF-36 across these amputation levels. Statistically significant differences were observed in several domains. The most prominent difference was found in the Role Emotional domain (FDR-adjusted *p* = 0.0179), followed by Role Physical (*p* = 0.0210), and Composite QoL Score (FDR-adjusted *p* = 0.0318). Participants with hip disarticulation and transfemoral amputation reported lower scores in these domains compared to those with transtibial amputation. Other domains such as Physical Functioning, General Health, and Emotional Well-being did not show statistically significant differences across groups. These findings suggest that level of amputation has a measurable but domain-specific impact on perceived quality of life.

### By composite QoL group

Participants were classified into Low QoL and High QoL groups based on the median composite QoL score. Categorical comparisons showed a statistically significant difference in gender distribution between the two groups (FDR-adjusted *p* = 0.025), with a greater proportion of females in the high QoL group. No statistically significant differences were observed across age group, education level, or amputation level after FDR adjustment. Table 3 provides the results of SF-36 domain scores by gender, age group, amputation level, and composite QoL group.

**Table 3:** Summary of SF-36 domain scores by gender, age group, amputation level, and composite QoL group. Mean and standard deviation (SD) values are presented for each subgroup, along with raw and FDR-adjusted *p*-values from Mann–Whitney *U* and Kruskal–Wallis tests. Group A and Group B refer to the two subgroups being compared in each row (e.g., for Gender: Group A = Male, Group B = Female; for age group: Group A ≤ 40, Group B ≥ 60). Statistically significant differences indicate group-level variation in specific SF-36 domains.

Comparison group	Domain	Group A (Mean ± SD)	Group B (Mean ± SD)	*p*-value	FDR adjusted *p*-value
Gender	Vitality	Male: 62.1 ± 18.2	Female: 66.5 ± 17.1	0.0086	0.0157
	Role Emotional	Male: 55.1 ± 42.7	Female: 61.7 ± 43.1	0.0172	0.0210
Age Group	Physical Functioning	<40: 61.7 ± 27.8	>60: 48.3 ± 22.0	<0.0001	<0.0001
	Role Physical	<40: 55.4 ± 38.3	>60: 43.2 ± 41.1	0.0025	0.0055
	Role Emotional	<40: 65.5 ± 44.1	>60: 48.4 ± 43.8	<0.0001	0.0002
	Pain	<40: 68.2 ± 20.1	>60: 58.1 ± 23.5	0.0001	0.0002
	Composite QoL	<40: 62.5 ± 13.1	>60: 55.9 ± 11.8	<0.0001	0.0002
Amputation Level	Role Physical	TT: 56.8 ± 41.5	TF: 47.5 ± 39.2	0.0163	0.0210
	Role Emotional	TT: 60.1 ± 42.3	TF: 51.3 ± 43.8	0.0114	0.0179
	Composite QoL	TT: 62.3 ± 12.7	TF: 58.0 ± 11.9	0.0318	0.0318
QoL Group	Gender	Male: 53.3% Low QoL	Female: 62.7% High QoL	0.0250	0.0275

### Multiple linear regression

Multiple linear regression analysis was conducted to examine the effects of age, gender, education level, and amputation level on each of the SF-36 domains and the composite QoL score. Age was significantly associated with lower scores for both Physical Functioning (*β* = –0.75, *p* < 0.001) and Role Physical (*β* = –0.56, *p* < 0.001), suggesting that increased age was associated with lower physical QoL. Gender was also significantly associated with Physical Functioning, with male participants scoring higher than females (*β* =  + 8.67, *p* = 0.0227). Education level and amputation level were not statistically significantly associated with most domains. The adjusted *R*^2^ values for the models ranged from 0.03 to 0.19, indicating modest explanatory power. Table 4 presents the multiple linear regression across SF-36 domains.

**Table 4:** Multiple linear regression across SF-36 domains. Regression coefficients (β) and *p*-values are reported for age, gender, education level, and amputation level as predictors across each SF-36 domain and the Composite QoL score. Adjusted *R*^2^ values indicate the proportion of variance explained by the model for each domain.

Domain	Age (β, *p*)	Gender (β, *p*)	Education (β, *p*)	Amputation level (β, *p*)	Adjusted *R*^2^
Physical Functioning	−0.75, **<** **0.001**	+8.67, **0.0227**	+2.02, 0.129	−1.13, 0.759	0.187
Role Physical	−0.56, **<** **0.001**	−4.15, 0.501	+3.46, 0.111	+7.59, 0.206	0.068
Role Emotional	−0.40, **0.0132**	−1.75, 0.782	+4.76, **0.021**	+9.41, **0.048**	0.086
Pain	−0.31, **0.0022**	+4.33, 0.456	+1.67, 0.380	+1.84, 0.656	0.051
Emotional Well-being	−0.15, 0.187	+2.77, 0.407	+3.14, **0.033**	+5.78, 0.142	0.029
Social Functioning	−0.19, 0.120	+1.79, 0.613	+2.29, 0.227	+4.53, 0.327	0.015
Vitality	−0.12, 0.268	+3.65, 0.273	+2.94, 0.184	+3.90, 0.401	0.019
General Health	−0.22, **0.048**	+0.95, 0.769	+0.45, 0.808	+1.68, 0.575	0.024
Composite QoL	−0.33, **<** **0.001**	+1.38, 0.427	+2.47, **0.031**	+3.85, 0.112	0.066

Bold values indicate statistically significant associations (*p* < 0.05).

To complement the significance testing in group comparisons, effect sizes were calculated to evaluate the magnitude of observed differences. For gender-based comparisons, the Mann–Whitney *U*-test revealed a small to moderate effect size for the Vitality domain (*r* = 0.20) and a smaller effect for Role Emotional (*r* = 0.15), despite both reaching statistical significance. Age-related differences were further examined using the Kruskal–Wallis test, with the largest effect observed in the Physical Functioning domain (*η*^2^ = 0.076), followed by Role Physical (*η*^2^ = 0.047), Role Emotional (*η*^2^ = 0.073), Pain (*η*^2^ = 0.059), and the Composite QoL score (*η*^2^ = 0.088), indicating small to moderate effect sizes. These findings suggest that while age and gender are statistically associated with QoL outcomes, the magnitude of these effects varies across domains and is generally modest.

In addition, a Spearman correlation analysis was conducted to explore the interrelationships among the eight SF-36 domains and the composite score. Strong positive correlations were observed between Physical Functioning and Role Physical (*ρ* = 0.57), and between Emotional Well-being and Vitality (*ρ* = 0.71), indicating that domains related to physical ability and emotional resilience tend to move together. The Composite QoL score correlated most strongly with Role Physical (*ρ* = 0.82), followed by Role Emotional (*ρ* = 0.72) and Vitality (*ρ* = 0.72), suggesting these domains are central contributors to overall perceived quality of life. The correlation matrix highlights an internal consistency among the SF-36 dimensions and reinforces the multidimensional nature of the instrument in capturing prosthetic users' experiences.

## Discussion

This study examined the quality of life (QoL) among lower limb prosthetic users in Jordan using the Arabic version of the SF-36 questionnaire. The results revealed a mixed pattern of outcomes. While participants reported relatively high scores in emotional and social domains, considerable impairments were observed in physical health–related domains, particularly physical functioning, general health, and role limitations due to physical health (Tables 2–4). These findings underscore the multifaceted nature of QoL among prosthetic users and align with the broader literature on amputation and rehabilitation outcomes (24–26).

The present study is consistent with earlier research conducted in Saudi Arabia (9), where lower limb amputees reported relatively preserved scores in emotional well-being and social functioning, while physical functioning and mobility were significantly compromised (Table 2). Similarly, research from Nepal using the SF-12 identified higher mental health scores relative to physical domains among prosthesis users, reinforcing the notion that emotional resilience and social integration may be sustained despite ongoing physical limitations (15). These cross-regional similarities may reflect common cultural and familial coping mechanisms in Middle Eastern and South Asian settings, such as strong family support systems and spiritual adaptation.

Recent work on disability in Jordan has emphasized that access to rehabilitation services, affordability of prosthetic components, and continuity of follow-up care are inconsistent, especially in public-sector clinics (27, 28). This context may help explain the moderate composite QoL scores observed in the current study. The use of a culturally adapted Arabic SF-36 provides an important foundation for future national evaluations and standardized patient outcome monitoring.

In terms of gender-related differences, group comparisons (Table 3) revealed that female participants scored significantly higher than males in the Role Emotional and Vitality domains. This contrasts with prior findings from both Middle Eastern and global studies, where women generally report lower QoL due to caregiving responsibilities and more restricted access to mobility resources (4, 9, 15, 29, 30). However, the regression analysis (Table 4) in the present study indicated that males scored higher in Physical Functioning after controlling for covariates. This suggests that gender differences may be domain-specific and context-dependent. One explanation could be that Jordanian women receive greater familial support or experience lower post-amputation work demands. Alternatively, sociocultural norms may influence how men and women express and report emotional well-being in self-assessments. These patterns may reflect culturally specific gender roles in Jordan, where men may face greater pressure to maintain physical functioning and return to work, while women often receive stronger familial and community support for emotional adjustment, as reported in regional rehabilitation studies (31, 32). These contrasting trends highlight the need for future qualitative research to explore gendered rehabilitation experiences and reporting behavior more deeply.

The divergence between domain-specific findings (Table 3) and the regression results (Table 4) suggested potential interaction effects, such as gender combined with age or amputation level. For instance, females reported higher scores in vitality and role-emotional domains, while males recorded higher scores in physical functioning after adjustment. These patterns indicated that gender influences may be context-dependent and moderated by other demographic or clinical variables.

Several age- and gender-related differences reached statistical significance (Tables 3, 4); however, the associated effect sizes were generally small to moderate. This finding indicated that while these demographic factors influenced quality of life, the magnitude of their practical impact was modest. For example, the gender-related difference in vitality (*r* = 0.20) (Table 3) represented a small but meaningful variation in perceived energy levels.

Age was a consistent negative predictor of physical QoL outcomes (Table 4). Older participants reported significantly lower scores in Physical Functioning, Role Physical, Pain, and Composite QoL domains. These findings are supported by previous studies from India, Iran, and Nepal, where older prosthesis users reported greater difficulty in ambulation, more comorbidities, and slower functional adjustment (6, 15, 33). Age-related declines in muscle strength, energy levels, and balance likely contribute to reduced prosthetic use and social reintegration. This reinforces the importance of targeted interventions for older adults, including physiotherapy, assistive devices, and tailored follow-up schedules.

While amputation level was significantly associated with several QoL domains in group comparisons (particularly Role Emotional, Role Physical, and Composite QoL, Table 3), it was not significantly associated with QoL in the multivariable regression analysis (Table 4). This finding differs from previous studies that reported poorer outcomes among individuals with transfemoral or hip disarticulation due to the greater energy demands and gait limitations associated with more proximal amputations (6, 9, 34). One possible explanation is that users with higher-level amputations in this cohort may have received more comprehensive prosthetic rehabilitation or psychosocial support, partially mitigating the expected negative effects. Alternatively, the limited number of hip and knee disarticulations in the sample (*n* = 4) may have reduced statistical power.

Level of education was not a significant predictor of QoL in this cohort (Table 4), despite literature suggesting a positive correlation between educational attainment and health outcomes. In contrast, studies from Saudi Arabia (9) and Pakistan (35) have shown that individuals with higher education report better QoL, potentially due to increased health literacy, better communication with clinicians, and higher expectations for rehabilitation. The lack of a significant education effect in the current study may be due to a relatively homogenous sample in terms of socioeconomic access to services or a cultural emphasis on familial care that buffers educational disparities. Other unmeasured confounding factors such as household income, digital literacy, and access to health information may also have contributed to this null finding (36).

Subgroup analysis comparing participants with low vs. high composite QoL scores confirmed a statistically significant difference only in gender, again favoring females. No significant differences were found for age group, education level, or amputation level. This suggests that traditional demographic and clinical characteristics may not fully account for differences in QoL among prosthesis users in Jordan, and that additional variables such as prosthetic comfort, community integration, peer support, and psychological adaptation are likely to play a role and should be explored in future mixed-methods or longitudinal studies.

This study has several strengths, including a large and demographically diverse sample, the use of a validated Arabic SF-36 instrument, and comprehensive statistical analysis incorporating descriptive, inferential, and multivariable approaches. However, some limitations should be noted. The cross-sectional design does not allow for causal inference, and the absence of variables related to prosthetic type (design), duration of use, cause of amputation, pain severity, mobility level, socioeconomic status, and rehabilitation intensity limits the ability to explore key clinical and contextual influences. Other important factors, such as prosthetic fit, long-term comfort, and device type, were also not captured, even though they are likely to influence physical quality-of-life domains. Similarly, strong family support systems and religious coping strategies common in Jordanian society may have contributed to the relatively high emotional well-being scores observed. Convenience sampling across 14 centers may have introduced selection bias, and those with limited access to rehabilitation services may be underrepresented. The very small size of certain subgroups (e.g., hip disarticulations, *n* = 3) reduces statistical power and these comparisons should be interpreted cautiously. The regression models explained only 3%–19% of the variance in QoL domains, suggesting that important unmeasured factors contribute to quality-of-life outcomes. Additionally, reliance on self-reported data introduces the possibility of recall bias and response bias, and cultural norms may have influenced how participants reported emotional well-being. Future research should incorporate longitudinal designs, probability sampling, and broader clinical variables to provide a more complete and causally interpretable understanding of quality of life among prosthetic users.

Finally, the regression models explained only a small proportion of variance in QoL domains (3%–19% adjusted *R*^2^), indicating limited explanatory power. This suggests that unmeasured clinical, psychosocial, and environmental factors likely contribute to quality-of-life outcomes.

This study represents one of the first comprehensive evaluations of QoL in lower limb prosthetic users in Jordan. While emotional and social health domains are relatively preserved, physical limitations remain prominent and are significantly associated with age and gender. These findings are consistent with regional research while highlighting contextual differences that may shape rehabilitation outcomes. They underscore the importance of holistic, culturally sensitive rehabilitation strategies that address both physical and psychosocial dimensions of post-amputation care.

Furthermore, by evaluating health-related quality of life in a vulnerable population within a low- to middle-income country, this study contributes to the global efforts aligned with Sustainable Development Goal 3: Good Health and Well-being. The findings support the need for inclusive, accessible rehabilitation services that promote equity and functional recovery for persons with disabilities in underserved settings.

## Conclusions

This study provides important insights into the quality of life of lower limb prosthetic users in Jordan. While emotional and social well-being domains were relatively preserved, significant impairments were observed in physical functioning and general health. Age and gender emerged as key factors associated with QoL, with older participants and males reporting lower scores in multiple domains. Although amputation level was not statistically significantly associated with QoL in multivariable regression, group comparisons revealed lower scores in role functioning and overall QoL among individuals with more proximal amputations. Education level did not show a significant association with QoL outcomes in either analysis.

These findings emphasize the importance of personalized, age-sensitive rehabilitation approaches and the integration of psychosocial support in prosthetic care. The results also underscore the utility of using multidimensional tools such as the SF-36 to capture the full spectrum of prosthetic users' experiences beyond physical recovery. Future research should build on these findings by incorporating longitudinal and mixed-methods designs to explore how clinical, functional, and contextual factors shape long-term QoL among amputees in Jordan and similar settings. Policy makers should consider expanding equitable access to prosthetic rehabilitation services, particularly in underserved regions, and clinical programs should integrate structured psychosocial support and culturally sensitive counseling for patients and families. Future research should test and refine rehabilitation models that incorporate these elements to improve both physical and emotional outcomes for prosthetic users in Jordan.

## Data Availability

The raw data supporting the conclusions of this article will be made available by the authors, without undue reservation.
